# Five-Fraction Proton Therapy for the Treatment of Skull Base Chordomas and Chondrosarcomas: Early Results of a Prospective Series and Description of a Clinical Trial

**DOI:** 10.3390/cancers15235579

**Published:** 2023-11-25

**Authors:** Morena Sallabanda, Juan Antonio Vera, Juan María Pérez, Raúl Matute, Marta Montero, Ana de Pablo, Fernando Cerrón, Mireia Valero, Juan Castro, Alejandro Mazal, Raymond Miralbell

**Affiliations:** Centro de Protonterapia Quironsalud, Pozuelo de Alarcón, 28223 Madrid, Spain; juan.vera@quironsalud.es (J.A.V.); juan.perezm@quironsalud.es (J.M.P.); alejandro.mazal@quironsalud.es (A.M.); raymond.miralbell@quironsalud.es (R.M.)

**Keywords:** chordoma, chondrosarcoma, hypofractionated, proton therapy

## Abstract

**Simple Summary:**

Chordomas and chondrosarcomas are rare and invasive tumors that frequently arise in the skull base. The extremely high local recurrence rate, due to the difficulty of achieving an optimal treatment with gross total resection, requires optimizing the efficacy of radiation therapy. The need for high radiation doses related to tumor radioresistance has motivated the application of proton therapy. Although this radiation modality, combined with dose escalation over 70 GyRBE, has shown the higher local control and survival results, the rate of local recurrence is still significant. Potential radiobiological and clinical advantages may be achieved by implementing extremely hypofractionated proton therapy regimens in selected patients with chordomas or chondrosarcomas of the skull base.

**Abstract:**

(1) Background: Our purpose is to describe the design of a phase II clinical trial on 5-fraction proton therapy for chordomas and chondrosarcomas of the skull base and to present early results in terms of local control and clinical tolerance of the first prospective series. (2) Methods: A dose of 37.5 GyRBE in five fractions was proposed for chordomas and 35 GyRBE in five fractions for chondrosarcomas. The established inclusion criteria are age ≥ 18 years, Karnofsky Performance Status ≥ 70%, clinical target volume up to 50 cc, and compliance with dose restrictions to the critical organs. Pencil beam scanning was used for treatment planning, employing four to six beams. (3) Results: A total of 11 patients (6 chordomas and 5 chondrosarcomas) were included. The median follow-up was 12 months (9–15 months) with 100% local control. Acute grade I–II headache (64%), grade I asthenia and alopecia (45%), grade I nausea (27%), and grade I dysphagia (18%) were described. Late toxicity was present in two patients with grade 3 temporal lobe necrosis. (4) Conclusions: Hypofractionated proton therapy is showing encouraging preliminary results. However, to fully assess the efficacy of this therapeutic approach, future trials with adequate sample sizes and extended follow-ups are necessary.

## 1. Introduction

Chordomas are rare tumors developing from the remnants of the embryonic notochordal tissue that, via a mechanism still unknown, may remain along the axial skeleton. The intracranial clivus is the second most frequent arising site (30%). The most common histological subtype (95%) is classic chordoma, characterized by slow growth and a low rate of distant metastases but an extremely high local recurrence rate [1,2]. Likewise, chondrosarcomas have a slow growth potential (grade I–II) with a propensity for local recurrence. They commonly arise in the skull base from primitive mesenchymal or cartilaginous matrix cells. However, advanced disease and/or histologically grade III chondrosarcomas have a high metastatic potential and poor prognosis [3,4,5]. Although the clinical aggressiveness of chordomas is considered to be higher than chondrosarcomas, their tendency to grow in similar anatomical locations and to locally recur has required the same therapeutic management [6,7,8,9].

Skull base chordomas and chondrosarcomas destroy the bone and infiltrate the adjacent soft tissue, with a difficult identification of tumor margins. Neural and vascular structures such as cranial nerves, brainstem, carotid, and basilar artery can be compressed and/or encased by the expanding tumor. The optimal treatment is gross total resection (endoscopic endonasal, open approach, or a combination), but achieving adequate oncologic margins in these complex locations is often challenging or impossible, and surgery alone has been associated with suboptimal local control. In this context, postoperative radiation therapy is necessary in most scenarios. Nonetheless, the degree of hypoxia in these tumors is hypothesized to correlate with an increased probability of radioresistance, local recurrence, and metastasis [10,11,12,13].

However, the adverse effects associated with such doses using conventional fractionated photon therapy motivated the application of charged particles such as protons. Until now, the most extensive results in terms of efficacy have been provided by proton beam radiation therapy (PBRT) and dose escalation over 70 GyRBE. One of the main PBRT advantages is related to the reduction of the dose to the healthy tissues due to its physical dose deposition characteristics, specially enhanced with pencil beam scanning (PBS) [7,12,14].

Despite the good results derived from the combination of standard PBRT and dose escalation, the overall 5-year survival rate is estimated around 83% for chordomas and between 75–97% for chondrosarcomas [14]. In addition, a large part of the relapses occur within the irradiation field, mostly related to inadequate dose coverage, in order to protect organs at risk. Nonetheless, in other scenarios, the recurrences occur despite the administration of full radiation doses, suggesting a biological resistance to the irradiation scheme [15]. In this context, Georgetown University described an α/β ratio value of 2.45 Gy for chordomas, and a similar ratio for chondrosarcomas has been assumed. Thus, the estimation of a low α/β ratio predicts improved outcomes due to the higher biological sensitivity to large doses per fraction in hypofractionated regimens [16,17].

Extremely hypofractionated regimens in the treatment of chordomas and chondrosarcomas have only been described with dedicated photon radiosurgery technology. On the other hand, the advantages of PBRT, such as dose homogeneity and integral dose reduction, predict theoretical benefits of the implementation of high-dose low-fraction PBRT in the treatment of large skull base target volumes. In addition, the advances implemented in recent years towards inverse and modulated planning, the reduction of lateral penumbra, the estimation of relative biological effectiveness (RBE), and image guided (IGRT) positioning allow better dose distribution and greater delivery precision. 

In this context, the purpose of our study is to describe the design of our phase II clinical trial on hypofractionated PBRT for these malignancies, and to present early results in terms of local control, acute, and late tolerance of the first prospective series of 11 patients.

## 2. Materials and Methods

### 2.1. Clinical Trial Design

A phase II clinical trial with a low level of intervention was designed and approved. The hypothesis of the study is to demonstrate that adjuvant or definitive PBRT in 5 fractions for selected patients with chordomas or chondrosarcomas of the skull base can lead to an improvement in local control and clinical tolerance compared to the published data of standard PBRT. The trial is currently ongoing and recruiting with the aim to achieve 20 enrolled patients [18]. The series we are presenting is from patients not included in the trial but evaluated prospectively. The trial is currently registered in ClinicalTrials.gov and this is the link (NCT05861245).

Consequently, the primary objectives of the study are the evaluation of the following:

- Acute and late toxicity according to Common Terminology Criteria for Adverse Events (CTCAE-v5) [19].
- One-, three-, five- and ten-year local control determined using magnetic resonance (MRI) with gadolinium and overall survival, comparing the results with historic standard PBRT cohorts.

On the other hand, the secondary objectives are as follows:

- The evaluation of the quality of life of the patients 3 months after the end of the treatment, using a specific questionnaire EORTC-QLQC30 and BN20.
- The evaluation of the dosimetric benefits using techniques that allow an improvement in the dose gradient, achieving a better coverage of the clinical target volume (CTV) and decreasing the dose in surrounding risk organs.

The therapeutic schemes that will be proposed to patients based on clinical criteria such as tumor size and relationship of the tumor with adjacent critical organs are the following:

- For chordomas: 37.5 GyRBE in 5 consecutive sessions of 7.5 GyRBE per fraction.
- For chondrosarcomas: 35 GyRBE in 5 consecutive sessions of 7 GyRBE per fraction.

The selected inclusion criteria for this study are as follows:

- Patients ≥ 18 years old.
- With a baseline classification on the Karnofsky performance status scale (KPS) ≥ 70%.
- With confirmed histological diagnosis of chordoma or chondrosarcoma of the skull base.
- With a maximum CTV of 50 cc.
- Whose relationship to organs at risk (OARs) allows compliance with the necessary dose restrictions to receive hypofractionated proton therapy in 5 fractions.

Patients included in the study must meet dosimetric parameters that include the following:

- Tumor CTV coverage of at least D95 > 90%.
- Correct compliance with the dose restrictions, at least in the nominal scenario, for critical organs (optic pathway, brain stem, and spinal cord) according to the guidelines published and available in the literature:
  ○ Dose constraints for 5 fractions [20,21,22]:
    - Optic nerves and chiasm: D0.03 cc ≤ 25 GyRBE, V23.5 < 0.5 cc.
    - Brainstem: D0.03 cc ≤ 31 GyRBE, V23 < 0.5 cc.
    - Spinal cord: D0.03 cc ≤ 30 GyRBE, V23 < 035 cc.
  ○ Nonmandatory but desirable dose constraints for 5 fractions [20,21,22]:
    - Right and left temporal lobe: V35 < 1.7 cc, V30 < 5.5 cc, V28 < 7 cc.
    - Right and left cochlea: D0.03 cc < 25 GyRBE

The defined exclusion criteria for this study are as follows:

- Patients with distant metastases.
- Patients who have received previous irradiation in the same location.
- Patients whose clinical or dosimetric characteristics do not meet the inclusion criteria.
- Patients who are simultaneously participating in another study that may affect the results of this protocol.

### 2.2. Clinical and Physical Aspects

Image acquisition and volume delineation is equivalent to conventional treatment:

- Computerized tomography (CT) is required to have a field of view of 32 cm and a matrix size of 500 × 500 voxels. The same acquisition with and without iodinated contrast is performed in the supine position with a thermoplastic mask for immobilization. The slice thickness is selected to be lower or equal to 1.25 mm, so enough spatial resolution is guaranteed.
- Thin-cut (1.25 mm) 3 Tesla MRI with 3D T1, T1 postgadolinium and T2 is obtained.
- In the case of previous surgery, the preoperative MRI will be fused.

Rigid and deformable registration is performed between the two planning images to ensure the proper delimitation of the critical structures:

- The GTV (gross tumor volume) is contoured including the macroscopic tumor based on CT and MRI with and without contrast.
- The CTV is contoured on MRI and CT (bone window), including the current GTV, the pre-surgical GTV considering the corresponding anatomical modifications after surgery (surgical bed), the surrounding areas suspected of containing microscopic disease (the relevant regions of the clivus, adjacent anatomical compartments at risk of direct spread such as cavernous sinus and the surgical approach) with a margin of 5 mm limited by anatomy. This CTV must respect anatomical boundaries, such as unaffected bone, dura mater, and cerebrospinal fluid.

Treatment planning is performed with intensity-modulated proton therapy (IMPT) employing 4 to 6 noncoplanar beams. The beam arrangement is selected in such a way that published recommendations related to beam separation are fulfilled [23]. Clinical Monte-Carlo algorithms are required to be employed for robust dose optimization and computation. The isotropic setup uncertainty is selected to be 1 mm, and the range uncertainty is established as a 3.5% of the range. Optimization objectives are selected to obtain dose distributions that can meet the dosimetric criteria of the trial. The maximum dose grid allowed for computation is 1.5 mm, as this is considered as the upper threshold for accurate dose computation. External personalized collimators or apertures are also considered in selected beams of every plan to improve dose fall-off and properly sharpen dose distributions if necessary. Linear energy transfer (LET) distributions are computed in a routine basis for all the plans in the study.

The plan evaluation and approval is achieved by the joint evaluation of a radiation oncologist and a medical physicist after discussion in an institutional PBRT board. In addition to verifying that all dosimetric criteria are met in the nominal plan, a robust evaluation in 28 discrete scenarios is performed for target and mandatory OARs. The LET distribution using the McNamara model [24] is also qualitatively and semi-quantitatively evaluated in the nominal scenario.

Adaptative radiotherapy (ART) strategies are required during the treatment courses of the patients to verify the accuracy of the planned dose distribution and the lack of anatomical changes that may have a dosimetric impact. In this sense, offline ART with three control scannes, where plan distribution can be recomputed and evaluated prior to treatment the first, third, and fifth session, are recommended. Also, image-guided radiotherapy (IGRT) is required to properly verify patient positioning during treatment delivery. In particular, daily CBCT images for patient setup and oblique images prior to every noncoplanar beam delivery are recommended.

### 2.3. Preliminary Cohort Results

#### 2.3.1. Description of the Prospective Cohort

Eleven consecutive patients with biopsy-proven chordomas and chondrosarcomas were prospectively included in a five-fraction PBRT protocol between June 2022 and January 2023. The inclusion and exclusion criteria and treatment dose are described in the study design. Information about age, gender, date of diagnosis and onset symptoms, tumor volume at diagnosis, date and type of surgery performed, grade of resection, postoperative symptoms, CTV volume, PBRT date, dosimetric characteristics (number of beams and apertures, CTV coverage, maximal dose to CTV and critical organs), acute tolerance at 0, 1, and 3 months and late tolerance at 6, 9, 12, and 15 months after treatment according to CTCAE-v5, and local control status according to 3-, 6-, 9-, 12-, and 15-month follow-up MRIs was collected in a data base and analyzed. 

#### 2.3.2. Statistical Analysis

For the descriptive analysis, frequency tables were created on qualitative variables. In quantitative variables, mean, range, and median were described. For the analysis of survival, the date of diagnosis and PBRT treatment to the date of appearance of the corresponding event or the date of the last follow-up was collected. Overall survival (OS), progression-free survival (PFS), and grade 3 toxicity-free survival were estimated using the Kaplan–Meier method. The confidence interval (CI) was designated as 95%.

## 3. Results

### 3.1. Descriptive Analysis of the Prospective Series

Eleven patients (six males and five females) with a median age of 42 years old (range 29–79 years old) and histologically confirmed classic chordomas (n = 6) or grade I-II chondrosarcomas (n = 5) of the skull base were prospectively included in a five-fraction PBRT protocol. The most common symptoms at diagnosis were headache and diplopia due to VI cranial nerve palsy (45%), followed by facial hypoesthesia related to trigeminal nerve compression (27%), III cranial nerve palsy, blurred vision, and nausea (18%). In addition, one patient experienced anosmia because of mass effect on lamina cribosa, one patient with an anterior tumor debuted with dysphagia and xerostomia, and one patient with a lower lesion experienced tetraparesis, secondary to a compression of the medullary junction. The median preoperative tumor volume on diagnostic MRIs was 20.7 cc (range 7.9–38.8 cc).

Ten patients had radical surgery with complete (n = 2) or subtotal resection (n = 8) and one patient had an excisional biopsy. Six patients improved or reversed symptoms after surgery, three patients remained clinically stable, and one patient had a brain hemorrhage developing a complete aphasia and left-body hemiparesis, that was recovering at the time of radiation. In addition, two patients had bacterial meningitis, secondary to a cerebrospinal fluid fistula, with adequate recovery prior to PBRT. The median KPS at the beginning of PBRT was 90% (range 70–100%). The median treatment time between surgery and radiation was 4 months (range 2–4.5 months).

### 3.2. Treatment Delivery Characteristics

**Figure 1:**
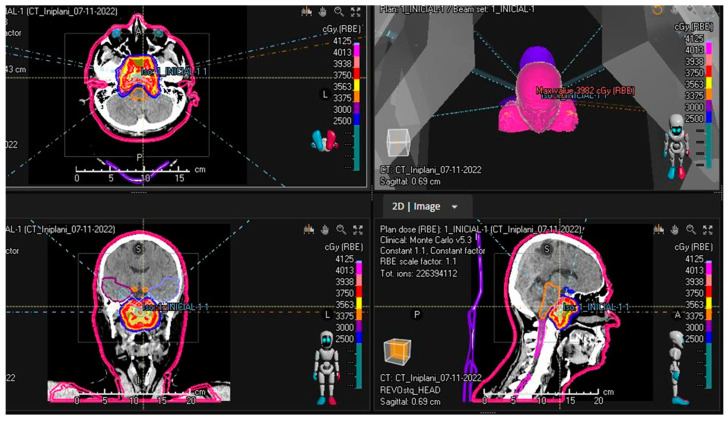
Dose distribution and beam orientation using six beams on a patient with a skull base chordoma treated with five-fraction PBRT.

The PBRT characteristics are described in detail in Table 1. Treatment was prescribed in five fractions, administered in consecutive days, with a weekend in-between. Most of the patients were treated using apertures to optimize dose gradient; two apertures were used in two different beams, per plan. All the plans complied with mandatory dose constraints, temporal lobe, and cochlea dose constraints, at least in the nominal scenario. It has not been necessary to create adaptive planes during any treatment course. In addition, Figure 1 shows the dosimetry of a patient with a skull base chordoma who received adjuvant hypofractionated PBRT.

### 3.3. Survival and Toxicity Analysis

The median follow-up from hypofractionated PBRT was 12 months (range 9–15 months). No local recurrences have been reported in the follow-up MRIs performed every 3 months after treatment. Acute toxicity during the first 3-month follow-up was mild, with grade I–II headache (64%), grade I asthenia (45%), reversible alopecia (45%), grade I nausea (27%), and grade I dysphagia (18%). One patient with chondrosarcoma showed nystagmus and mild loss of strength in the left lower limb related with an ischemic brainstem lesion, in a low dose region, not clearly radiation-related, and another patient with chordoma developed IV cranial nerve palsy. These symptoms appeared 3 months after treatment in both patients and reversed after a short course of steroids. 

In addition, two patients developed grade 3, symptomatic, temporal lobe necrosis, 9 and 12 months after treatment. One patient with a chordoma, who had a postsurgical meningitis, developed radiological bilateral necrosis. The other patient with a chondrosarcoma had radiological signs of necrosis in the right temporal lobe. Both patients developed seizures and needed hospitalization. After steroid and antiepileptic medication, both patients had good response and returned to normal activity. After evaluating the dosimetry of both treatments, the physical dose constraints were accomplished in the nominal scenario, but there seemed to be a correlation with the LET distribution, currently under investigation in our center. A detailed description of toxicity is shown in Table 2.

## 4. Discussion

Scrupulous attention and significant expertise are required for successful irradiation in these challenging cases. Although standard PBRT has proven to produce the best results, there still is a significant rate of long-term local recurrence rates with an impact on overall survival. In addition, the limited availability of these facilities, together with the administration of very long treatment schedules, remains an obstacle to the widespread adoption of this technique as a therapeutic standard [25,26,27].

Due to all these limitations and the important advances in terms of precision and dose distribution, the concept of hypofractionation has gained weight within radiation oncology thanks to its potential benefits in cost-effectiveness results with similar or superior local control rates. The first publications on the hypofractionated treatment of chordomas, mainly of the skull base, go hand in hand with photon radiosurgery systems, using dedicated equipment such as the GammaKnife or CyberKnife. In the last 15 years, single-dose or hypofractionated treatment schemes have been explored as a therapeutic alternative to escalate the dose, improve the protection of organs at risk, and reduce treatment time [5,8,9,16,28,29,30,31,32,33,34].

Studies on single-fraction photon stereotactic radiosurgery (SRS) show results comparable to PBRT with five-year overall survival rates up to 86% in chondrosarcomas and 80% in chordomas when ≥15 Gy were prescribed and volumes ≤ 7 cc were treated. In this context, it is essential to emphasize that tumor volumes usually treated in chordomas and chondrosarcomas of the skull base exceed, in most cases, this volume limit, since the entire clivus must be included in order to reduce the risk of marginal recurrence. For this reason, hypofractionated photon stereotactic radiotherapy (HFSRT) has theoretical advantages in larger volumes. Several publications evaluate the role of HFSRT, mostly in five fractions with a prescription dose of 24–43 Gy, depending on histology and therapeutic setting (radical, adjuvant, or reirradiation). Although the available evidence includes single-institution small series and limited follow–up, the best overall survival results at 3 and 5 years obtained were 90% and 74% for chordomas, respectively, and 100% for chondrosarcomas. The most widely used regimen was 37.5 Gy in five fractions for chordomas and 35 Gy in five fractions for chondrosarcomas. The long-term toxicity reported in most of these studies does not register worse data than those published with conventional fractionation PBRT when it comes to primary treatments, but the description of toxicities in most of these series is rather poor due to clinical heterogeneity and retrospective evaluation [5,8,9,16,28,29,30,31,32,33,34].

It seems clear that hypofractionation exerts varying influences on each of the major clinical end points of radiotherapy studies: acute toxicity, late toxicity, and local control. These differences will depend on radiation volumes, conformity, and dose gradient, but also on specific tumors and healthy tissue radiobiology. Acute toxicity is one of the most straightforward clinical outcomes to consider. Hypofractionated regimens will commonly alter overall treatment time and total dose. In this regard, the total dose reduction could have an important impact on improving acute tolerance [35]. On the other hand, there is a clearly described increase in lethal DNA damage when hypofractionated regimens are used in tumors with low α/β ratio, such as chordomas and chondrosarcomas, among other biological effects such as an increased immune response and endothelial cell apoptosis. These mechanisms act synchronously with DNA damage, increasing tumor cell death. Finally, the impact on long-term toxicity is the most feared consequence of extending and standardizing the use of these therapeutic regimens. The possibility of increasing the risks of optic neuropathy and brainstem necrosis is one of the most important issues, conditioning CTV coverage. Nonetheless, there is strong evidence concerning dose limits and the risk of adverse events with hypofractionated regimens due to the extensive number of publications on a variety of skull base and perioptic tumors commonly treated in five fractions. Also, the panhypopituitarism, temporal lobe necrosis, and cranial nerve neuropathy are late adverse events described in the literature after standard PBRT, but since the α/β ratio of chordomas is similar to the α/β ratio of healthy brain structures at risk, the dose-dependent toxicity should not substantially differ in different fractionation regimens [36,37,38].

Regarding accessibility to PBRT therapy, it should be mentioned that, today, it remains a limited resource, with only 113 facilities in operation worldwide in 2023 [39], of which two new centers are in Spain, where the first center has been operating since 2019. In addition, many patients must travel to access this technology, so reducing the time a patient is away from home and their support network can have significant financial and psychosocial implications. That is why the implementation of hypofractionation within PBRT has gained weight in the last 10 years, increasing the number of publications in this regard, which reflects the interest in this treatment approach [40].

Regarding dosimetric characteristics, Cao et al. presented a dosimetric study comparing different hypofractionated stereotactic treatment schemes in the treatment of intracranial tumors > 3 cm in greatest diameter. Treatment plans with GammaKnife, Cyberknife, and VMAT were generated and compared with PBRT plans. The authors suggested that proton therapy represents a desirable alternative to advanced photon techniques for treating large, irregularly shaped volumes close to critical structures, such as chordomas and chondrosarcomas [41].

In connection with the adoption of new dose per fraction schemes in PBRT, the potential uncertainty in RBE values should be carefully evaluated. In the case of hypofractionation, as the dose per fraction increases, a decrease in the RBE of PBRT could be expected, due to the shape of the cell survival curves for protons and photons (at larger single doses, the gradients of the cell survival curves are more equivalent). In addition, experimental studies suggested a RBE increase with LET, for 2 GyRBE per fraction irradiations, registering higher values (70%) in distal fall-off of a spread-out Bragg peak, but for 6 GyRBE irradiations, the RBE decreased by up to 10% [37,42,43,44]. However, caution should be used when introducing these new hypofractionanted schemes in clinical practice, and patient monitoring is required due to the increase of uncertainty in RBE values. 

Our preliminary results of this first prospective series cannot be evaluated with statistical significance, because of the limited cohort that has been analyzed. In terms of local control, the modest follow-up prevents from reaching any conclusions regarding therapeutic efficacy. Regarding acute tolerance during the first 3-month follow-up, mild grade 1–2, reversible symptoms have been described, despite the previous radical surgery, the large treatment volumes, and the high doses. On the other hand, regarding late adverse events, two patients experienced grade 3 temporal lobe necrosis. Available data on standard PBRT of skull base tumors show a 15% risk of any grade temporal lobe necrosis after 3 years, pointing to a slightly lower risk compared to our series, but this comparison may be biased by the low statistical significance level in our data due to the limited number of patients included in the analysis [37]. To the best of our knowledge, this is the first ongoing clinical protocol evaluating hypofractionated PBRT in the base of the skull for these malignancies, so strong clinical outcomes in terms of survival, local control, and normal tissue complication probability should only be compared to current standard techniques of treatment with special care to avoid possible biases due to patient selection among other possible causes.

The preliminary analysis of our data shows that attention should be paid to dose and LET evaluation in the temporal regions. In particular, beam arrangements with six or five beams instead of four have shown potential to decrease LET and dose values in these areas, avoiding new temporal lobe necrosis events. However, due to the limited statistical significance of our data, conclusions cannot be derived in this sense yet. Clear guidelines for LET computation and evaluation are still missing in current clinical practice for all kinds of PBRT treatments. We fully support the efforts taking place in the PBRT community and all the proposals bringing light to the understanding of these LET-related effects. In this study, we decided to compute LET distributions for all patients and evaluate these distributions paying special attention to the regions that received more than 50% of the prescription dose, with a LET higher than a threshold value of 5 KeV/µm, and the organs at risk where these values took place. It should be finally mentioned that LET analyses have never conditioned plan dose distributions; instead, the combination of an appropriate fulfilment of the dose criteria and a favorable LET evaluation seems to be the optimal approach.

Finally, we would like to mention the increasing efforts that have been taking place in research regarding the field of molecular biology, immunology, nanomedicine, and interventional radiology to improve overall survival, particularly when standard local therapy fails [44,45,46].

## 5. Conclusions

The prospective evaluation of extremely hypofractionated PBRT regimens is a fundamental and necessary challenge today for the treatment of low α/β, radioresistant, large intracranial tumors such as chordomas and chondrosarcomas. In this context, we describe the first clinical trial of five-fraction PBRT for the treatment of these malignancies. The combination of high precision hypofractionated treatment schemes with the dosimetric advantages of PBRT is being evaluated with acceptable early tolerance in a small prospective series, but careful considerations of biological dose uncertainties should be made in order not to increase the risk of late adverse events. Adequate enrollment and late follow-up results of this clinical trial will provide strong evidence regarding this matter. 

## Figures and Tables

**Figure 2:**
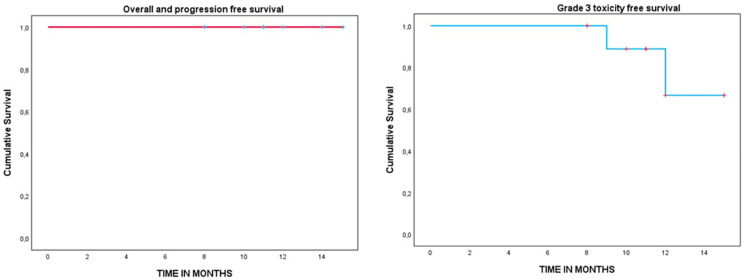
Kaplan–Meier survival analysis curve of OS, PFS, and grade 3 toxicity-free survival.

**Table 1:** Hypofractionated PBRT characteristics.

	Chordomas (n = 6)	Chondrosarcomas (n = 5)
Total dose	37.5 GyRBE	35 GyRBE
Dose per fraction	7.5 GyRBE	7 GyRBE
Median CTV (range)	32.2 cc (13.7–43.5 cc)	32.5 cc (10.6–47.5 cc)
Median number of beams (range)	4 (4–6)	4 (4–5)
Use of apertures	4 patients	3 patients
Median CTV coverage (range)	D95 = 95% (92.2–99.8%)	D95 = 96% (94.2–98.7%)
Median CTV D99 (range)	39 Gy (39.9–38.8 GyRBE)	36.4 Gy (37–36 GyRBE)
Median D0.03 cc at brainstem (range)	26 Gy (30–24.7 GyRBE)	26.5 Gy (29.1–25.2 GyRBE)
Median D0.03 cc at optic pathway (range)	20.2 Gy (23–8.9 GyRBE)	20.7 Gy (23.6–16.3 GyRBE)

**Table 2:** Description of toxicity.

	Chordomas (n = 6)	Chondrosarcomas (n = 5)
Acute toxicity (0–3 months)		
Headache grade I–II	4 patients (67%)	3 patients (60%)
Asthenia grade I	3 patients (50%)	2 patients (40%)
Reversible alopecia	2 patients (33%)	3 patients (60%)
Nausea grade I	1 patient (17%)	2 patients (40%)
Dysphagia grade I	2 patients (33%)	
Subacute toxicity (3–6 months)		
IV cranial nerve palsy	1 patient (17%)	
Late toxicity (6–15 months)		
Temporal lobe necrosis grade III	1 patient (17%)	1 patient (20%)

Kaplan–MeierOS, PFS, and grade 3 toxicity-free survival curves were obtained and are shown in Figure 2. The actuarial median follow-up from hypofractionated PBRT was 12 months (95% CI 10.7–13.5 months) with an OS and PFS of 100%. The actuarial grade 3 toxicity-free survival at 12 months was 72.7% (95% CI 70.9–74.5%).

## Data Availability

Data are unavailable due to privacy and ethical restrictions.
